# Policy and strategies addressing prevention and control of antimicrobial resistance in Brazil: A scoping review protocol

**DOI:** 10.1371/journal.pone.0263305

**Published:** 2022-01-28

**Authors:** Joslaine de Oliveira Nunes, Raissa Allan Santos Domingues, Roberto Mauro Pinto Coelho Barcellos, Bárbara Manuella Cardoso Sodré Alves, Isis Polianna Silva Ferreira de Carvalho, Noemia Urruth Leão Tavares

**Affiliations:** 1 Program in Collective Health, Faculty of Health, University of Brasília (UnB), Brasilia, Brazil; 2 Brazilian Ministry of Health, Brasília, Distrito Federal, Brazil; 3 Center for Tropical Medicine, Faculty of Medicine, UnB, Brasília, Brazil; 4 Program in Pharmaceutical Sciences, Unb, Brasília, Distrito Federal, Brazil; 5 Department of Pharmacy, Faculty of Health Sciences, UnB, Brasilia, Brazil; Babol University of Medical Science, ISLAMIC REPUBLIC OF IRAN

## Abstract

**Introduction:**

Antimicrobial resistance (AMR) is considered one of the biggest health challenges of the 21st century. It has both social and economic consequences; therefore, timely review of public health policies that have been designed to manage AMR is essential. Brazil too has developed and implemented various polices for the prevention and control of AMR. However, till date, no study provides insights regarding the various public health policies or other programs implemented by Brazilian institutes.

**Objective:**

The objective is to define a scoping review protocol of policies that were developed to address prevention and control of antimicrobial resistance in Brazil, from a human health perspective.

**Method:**

This protocol has been registered in the Open Science Framework (DOI 10.17605/OSF.IO/EC9ZJ). Indexed literature in English, Spanish and Portuguese published till December 2020 in Lilacs, PubMed, Embase, and official websites of the Brazilian government will be reviewed. This review considers all studies identified through a comprehensive search of peer-reviewed and grey literature databases that have a reference for policies made for managing AMR in Brazil. The criteria for the scoping review will be set by two evaluators. A third evaluator will be consulted, if there is any disagreement between the two primary evaluators. A standardized form will be used for data extraction from the selected studies. The results will be presented in a tabular form with narrative abstracts related to the topics identified through the scoping review protocol. The PRISMA extension for Scoping Reviews tool will be used.

## Introduction

Antimicrobial resistance (AMR) occurs when microorganisms (e.g., bacteria, viruses, parasites and fungi) develop the capacity to adapt and grow in the presence of substances that were capable of eliminating them [1]. This phenomenon is becoming both an economic and social concern because of AMR’s ability to rapid disseminate, which poses a risk to human health, animals, and plants; and the emergence of resistance to new drugs, leaving no therapeutic alternative and thus becoming a lethal threat to living beings [2, 3].

AMR is now recognized as a global human health problem by the World Health Organization (WHO), Food and Agriculture Organization (FAO), the World Organization for Animal Health (OIE), World Bank, and other institutions of international relevance [3, 4]. An economic report projects that by 2050, AMR will cause 10 million deaths annually and a reduction of 2 to 3.5% in the Gross Domestic Product (GDP), costing the world an upwards of 100 trillion US dollars [5].

To overcome this threat, in 2015, the WHO published the Global Action Plan on Antimicrobial Resistance, at its 68th World Health Assembly. It impelled its member states to write their own national plans in accordance to the Global Action Plan [6]. In 2018, Brazil published its *Nation Plan on Prevention and Resistance Control to Antimicrobials on the Scope of One Health 2018–2022 (PAN-BR)* [7].

The history and evolution of public policies to tackle AMR are well documented by the United Kingdom, France, Sweden, and Denmark [8–11]. However, in Brazil, there are no published studies in indexed journals that review the available evidence of AMR management in accordance with the Nation Plan.

It is important to bridge this gap and collate evidence of action against AMR. Considering that mapping of available evidence can be ideally done through the scoping review method [12, 13], this study employs a scoping review protocol. The review was idealized to identify and document the history of the Brazilian government’s action and other actors’ strategies to contain AMR as well as its involvement in relevant policy development at both national and international level.

We emphasize that a search (in December 2020) of Cochrane Database of Systematic Reviews, PubMed and Epistemonikos, produced no scoping reviews on prevention and control of AMR in Brazil. It is hoped that this study can contribute literature for future scientific studies.

### Review question

What are the various policies and strategies, from a human health perspective, that exist in Brazil for prevention, surveillance, and control of antimicrobial resistance? Mainly, what has been done about this issue in Brazil and what are the gaps in implementation and research?

## Methods and analysis

The aim of the paper is to define a review protocol of policies and strategies that were developed to address prevention and control of antimicrobial resistance in Brazil, from a human health perspective.

This protocol employed a scoping review method [12]. This review method uses the JBI guidelines [13] and the Preferred Reporting Items for Systematic Reviews and Meta-Analysis Extension for Scoping Reviews (PRISMA-ScR) [14]. Recommendations from other published articles on scoping review methodology were also considered [15–18]. This protocol has been registered in the Open Science Framework (DOI 10.17605/OSF.IO/EC9ZJ).

### Eligibility criteria

To define the eligibility criteria for the review, factors such as population, concept, and context, were established in accordance with the Joanna Briggs Institute Reviewers Manual [13]. These factors, or terms, are summarized and further described in Table 1.

**Table 1:** Population, concept, and context for the review question.

Term	Definition
Question	Which policies and strategies were developed for the prevention, surveillance, and control of antimicrobial resistance, from the human health perspective, in Brazil?
Population	Humans
Concepts	Policy: plans, programs, projects, databases or information systems, normative or regulatory actions, defining rules, directives, and standards or incentives developed by the government or Brazilian institutions in partnership with international institutions or on their own [19–21].
	Strategies: national and international AMR monitoring and surveillance networks, studies on AMR treatment and diagnosis and stewardship programs developed by Brazilian institutions in partnership with international institutions or on their own.
	Antimicrobial resistance: resistance developed by bacteria, fungi, viruses, and protozoans to the medicines developed to eliminate them [1].
Context	Policies at the national, state, and institutional levels.

### Concept

The concept of AMR policies is very broad [19]. This scoping review protocol considers (i) policies as all plans, programs, projects, databases or information systems, research, and normative or regulatory actions, defining rules, directives, and standards or incentives [19–21] at both federal and state level, and (ii) strategies as (a) AMR international surveillance networks with participants from one or more Brazilian, public or private health institutions, (b) national or international multicenter studies about treatment or diagnostic of resistant microorganisms, carried out in collaboration with one or more Brazilian health institutions, (c) stewardship programs developed by Brazilian institutions.

The concept of AMR will be verified in accordance with the content of Table 1. Additionally, all studies that contained the terms highlighted as concept items, such as “international surveillance,” “networks,” “programs,” “multicenter studies,” and “stewardship programs,” in the title, abstract, or keywords were included for full reading.

### Context

The context of the concepts classified in the previous section are as follows: item (i) includes policies developed by federal or state governments and item (ii) comprise strategies developed by one or more Brazilian health institutions, public or private.

The scoping review will include all studies published before December 2020, in either English, Spanish or Portuguese, and will consider all study designs that have a reference for policies and strategies made for managing AMR in Brazil.

Studies will be excluded if they are not conducted in Brazil or in partnership with brazilian institutions or if they are literature reviews describing the concept of AMR without interventions. Studies will also be excluded if they estimated the burden of AMR without describing interventions used to address it.

### Search strategy

For this study, an electronic search was carried out using keywords and synonyms pertaining to AMR. For example, “antibiotic resistance,” “antiviral resistance,” “antifungal resistance,” and “antimicrobial stewardship,” in tandem with restrictive operators like “Brazil.” The full electronic search strategy from each database is presented in S1 Appendix.

### Information sources

Search for information was carried out electronically on the following databases -PubMed, Embase, and Lilacs. The search for gray literature was performed on Brazilian government websites, as the Ministry of Health (https://www.gov.br/saude/pt-br) and the National Health Surveillance Agency–Anvisa (https://www.gov.br/anvisa/pt-br).

### Study selection

The selection process for literature to be included in the scoping review consists of three stages:

1. Exclusion of repeated articles: all identified registries will be imported to Zotero [22] for bibliography management and exclusion of duplicates.
2. Analysis of all article titles followed by an abstract review: web app Rayyan [23] will be used at the study selection stage.
3. Complete analysis of selected articles: if a study is unavailable, evaluators will contact its corresponding author.

The second and third stages will be carried out by two evaluators separately. If there is a disagreement, a third evaluator will be consulted. The decision process is presented as a PRISMA fluxogram [14]. The fluxogram will show the number of studies initially identified (databases and additional sources, where applicable), the elimination of duplicates, and the study selection stages. The fluxogram also presents reasons for removing or retaining an article.

### Data extraction

Data will be gathered from accepted articles by two independent evaluators. They will use standardized forms that comply with the objectives of this study. Any disagreement between evaluators will be resolved through discussions.

For data extraction, a standardized form was developed specifically for each concept topic charted in Table 1. The list and definition of all variables from which the data will be extracted is presented in S2 Appendix.

## Results

Results will be presented in a tabular form, combined with a descriptive abstract about each of the topics identified through the scoping review protocol. A table will be drafted to compile all extracted data, grouped by study type. A map and timeline will be created for better result presentation. The results will focus on policies, strategies and research from the Brazilian point of view, and internationally, when applicable.

## Discussion

This study dicusses the temporal evolution of the policies and strategies that emerged in Brazil. Special focus will be given to policies developed after the publication of the Global Action Plan in 2015, aiming to verify the fulfillment of the objectives proposed by WHO to its member states. Whenever possible, results will be discussed in comparison with those of studies from other countries in Latin America and other regions. The authors consider that a strength of the study lies in its breadth, since no study so far has mapped so many Brazilian policies for the prevention and control of REAM. One of the study’s limitations is the fact that a broad search was performed, due to the possibility that original studies on such policies did not provide explicit terms and phrases of interest in their titles, abstracts and keywords.

## Supporting information

S1 Appendix Full electronic search strategy from each database. (DOC)

S2 Appendix Form for data collection. (DOC)

S1 Checklist (DOCX)
