# Supplementary material for: Policy and strategies addressing prevention and control of antimicrobial resistance in Brazil: A scoping review protocol
Source: PLoS One. 2022 Jan 28;17(1):e0263305. doi: 10.1371/journal.pone.0263305 (PMC8797233; doi:10.1371/journal.pone.0263305)
Supplement: S1 Appendix — (DOC) [file pone.0263305.s001.doc]

**S1 Appendix. Full electronic search strategy from each database.**

Search date: December 22, 2020

| Pubmed | |
| --- | --- |
| (((((((((((((((((((((((((((((("Drug Resistance, Bacterial") OR ("Antibacterial Drug Resistance")) OR ("Antibiotic Resistance, Bacterial")) OR ("Drug Resistance, Microbial")) OR ("Drug Resistances, Microbial")) OR ("Antimicrobial Drug Resistance")) OR ("Antimicrobial Drug Resistances")) OR ("Antibiotic Resistance, Microbial")) OR ("Antibiotic Resistance")) OR ("Resistance, Antibiotic")) OR ("Drug Resistance, Multiple, Fungal")) OR ("Multidrug Resistance, Fungal")) OR ("Multidrug Resistance, Fungal")) OR ("Multiple Antifungal Drug Resistance")) OR ("Drug Resistance, Fungal")) OR ("Antifungal Drug Resistance")) OR ("Resistance, Antifungal Drug")) OR ("Antibiotic Resistance, Fungal")) OR ("Drug Resistance, Multiple, Viral")) OR ("Multiple Antiviral Drug Resistance")) OR ("Drug Resistance, Multiple, Bacterial")) OR ("Multiple Antibacterial Drug Resistance")) OR ("Drug Resistance, Viral")) OR ("Drug Resistances, Viral")) OR ("Antiviral Drug Resistance")) OR ("Antiviral Drug Resistances")) OR ("Antimicrobial Stewardship")) OR ("Stewardship, Antimicrobial")) OR ("Antibiotic Stewardship")) OR ("Stewardship, Antibiotic")) AND (("Brazil") OR ("Brazilian")) | 4.255 results |
| Embase |  |
| ('antibacterial drug resistance':ti,ab,kw OR 'antibacterial resistance':ti,ab,kw OR 'antibiotic non-susceptibility':ti,ab,kw OR 'antibiotic nonsusceptibility':ti,ab,kw OR 'antimicrobial drug resistance':ti,ab,kw OR 'antimicrobial resistance':ti,ab,kw OR 'bacterial drug resistance':ti,ab,kw OR 'bacterial resistance':ti,ab,kw OR 'bacterium resistance':ti,ab,kw OR 'drug resistance, bacterial':ti,ab,kw OR 'drug resistance, microbial':ti,ab,kw OR 'microbial drug resistance':ti,ab,kw OR 'antibiotic resistance':ti,ab,kw OR 'antiviral resistance':ti,ab,kw OR 'antifungal resistance':ti,ab,kw OR 'antifungal drug resistance':ti,ab,kw OR 'antimicrobial stewardship':ti,ab,kw OR 'antimicrobial stewardship program':ti,ab,kw) AND ('brazil'/exp OR 'brazil' OR 'brazilian'/exp OR 'brazilian') AND [1966-2020]/py | 2.560 results |
| Lilacs |  |
| (Antimicrobial Stewardship) OR (Programas de Optimización del Uso de los Antimicrobianos) OR (Gestão de Antibacterianos) OR (Gestão de Antibióticos) OR (Manejo de Antibacterianos) OR (Manejo de Antibióticos) OR (Manejo de Antimicrobianos) OR (Programas de Otimização de Antibióticos) OR (Programas de Otimização de Antimicrobianos) OR (Programas de Otimização de Uso dos Antibióticos) OR (Programas de Otimização do Uso de Antimicrobianos) [Palavras] or (Drug Resistance, Microbial) OR (Antibiotic Resistance) OR (Antibiotic Resistance, Microbial) OR (Antimicrobial Drug Resistance) [Palavras] and (Brazil) OR (Brazilian) OR (Brasil) OR (Brasileiro) OR (Brasileiros) OR (Brasileira) OR (Brasileiras) [Palavras] | 531 results |
