# Supplementary material for: Policy and strategies addressing prevention and control of antimicrobial resistance in Brazil: A scoping review protocol
Source: PLoS One. 2022 Jan 28;17(1):e0263305. doi: 10.1371/journal.pone.0263305 (PMC8797233; doi:10.1371/journal.pone.0263305)
Supplement: S2 Appendix — (DOC) [file pone.0263305.s002.doc]

**S2 Appendix. Form for data collection.**

| **Variable** | **Variable definition** | **i) policies at the federal or state government level** | **ii) international antimicrobial resistance surveillance programs or networks** | **iii) national and international multicenter studies** | **iv) nationwide surveillance studies** | **v) stewardship programs developed in Brazil** |
| --- | --- | --- | --- | --- | --- | --- |
| **Bibliographical details:** | Paper characteristics:  - article title  - authors  - year  - source (journal, internet address) | X | X | X | X | X |
| **Objectives** | Main objectives | X | X | X | X | X |
| **Type of study** | Methodological design | X | X | X | X | X |
| **Period** | Study data collection period | X | X | X | X | X |
| **Funding** | what is the funding source for the study? It is private or public funding? | X | X | X | X | X |
| **Countries** | countries participating in the study |  | X | X |  |  |
| **Brazilian institutions** | Brazilian institutions that carried out the research, partially or totally | X | X | X | X | X |
| **Brazilian states** | Brazilian states where the institutions that carried out the study are located | X | X | X | X | X |
| **Intervention** | Action or procedure performed with the objective of diagnosing or treating a clinical problem or action at the government level in order to solve a public health problem. | X |  | X |  | X |
| **Professionals involved** | Professionals responsible for carrying out the intervention. | X |  |  |  | X |
| **Setting** | level of care at which the study is carried out.  (Outpatient, hospital) | X |  |  | X | X |
| **Population** | It is the population from which the data comes and for which interference is made | X |  | X |  | X |
| **Population size** | Number of people included in the study population |  |  | X |  | X |
| **Sample type** | Type of biological sample analyzed qualitatively or quantitatively |  | X | X | X |  |
| **Sample size** | number of biological samples analyzed in the study |  | X | X | X |  |
| **Microorganism** | bacteria, fungi, viruses, or protozoa analyzed by microbiological or molecular techniques | X | X | X | X | X |
| **Susceptibility test** | Which antimicrobial susceptibility test method was performed in the study? |  | X | X | X |  |
| **drugs used in the susceptibility test** | list of drugs included in the antimicrobial susceptibility test |  | X | X | X |  |
| **Molecular biology** | description of the molecular biology method for identifying microorganisms |  | X | X | X |  |
| **Results** | main results of the analyzed study, according to the objectives established in the paper | X | X | X | X |  |
