# Supplementary material for: Policy and strategies addressing prevention and control of antimicrobial resistance in Brazil: A scoping review protocol
Source: PLoS One. 2022 Jan 28;17(1):e0263305. doi: 10.1371/journal.pone.0263305 (PMC8797233; doi:10.1371/journal.pone.0263305)
Supplement: S1 Checklist — (DOCX) [file pone.0263305.s003.docx]

**Preferred Reporting Items for Systematic reviews and Meta-Analyses extension for Scoping Reviews (PRISMA-ScR) Checklist**

| **SECTION** | **ITEM** | **PRISMA-ScR CHECKLIST ITEM** | **REPORTED ON PAGE #** |
| --- | --- | --- | --- |
| **TITLE** | | | |
| Title | 1 | Identify the report as a scoping review. | Yes. The title is "Policy addressing prevention and control of antimicrobial resistance in Brazil: a  scoping review protocol" |
| **ABSTRACT** | | | |
| Structured summary | 2 | Provide a structured summary that includes (as applicable): background, objectives, eligibility criteria, sources of evidence, charting methods, results, and conclusions that relate to the review questions and objectives. | Yes. The abstract is on page 2 of the manuscript. |
| **INTRODUCTION** | | | |
| Rationale | 3 | Describe the rationale for the review in the context of what is already known. Explain why the review questions/objectives lend themselves to a scoping review approach. | Yes. The review questions/objectives are on page 4 of the manuscript. |
| Objectives | 4 | Provide an explicit statement of the questions and objectives being addressed with reference to their key elements (e.g., population or participants, concepts, and context) or other relevant key elements used to conceptualize the review questions and/or objectives. | Yes. The key elements (e.g., population or participants, concepts, and context) are on pages 6-8 of the manuscript. |
| **METHODS** | | | |
| Protocol and registration | 5 | Indicate whether a review protocol exists; state if and where it can be accessed (e.g., a Web address); and if available, provide registration information, including the registration number. | Yes. The protocol was registered in the Open Science Framework (DOI 10.17605 /  OSF.IO / EC9ZJ). |
| Eligibility criteria | 6 | Specify characteristics of the sources of evidence used as eligibility criteria (e.g., years considered, language, and publication status), and provide a rationale. | Yes. The eligibility criteria is on page 5 of the manuscript. |
| Information sources* | 7 | Describe all information sources in the search (e.g., databases with dates of coverage and contact with authors to identify additional sources), as well as the date the most recent search was executed. | Yes. The information sources are on page 5 of the manuscript. The date of the most recent search is on Appendix 1. |
| Search | 8 | Present the full electronic search strategy for at least 1 database, including any limits used, such that it could be repeated. | Yes. The full electronic search strategy is on Appendix 1. |
| Selection of sources of evidence† | 9 | State the process for selecting sources of evidence (i.e., screening and eligibility) included in the scoping review. | Yes. The process for selecting sources of evidence is on page 6. |
| Data charting process‡ | 10 | Describe the methods of charting data from the included sources of evidence (e.g., calibrated forms or forms that have been tested by the team before their use, and whether data charting was done independently or in duplicate) and any processes for obtaining and confirming data from investigators. | Yes. The methods of charting data are on page 8. |
| Data items | 11 | List and define all variables for which data were sought and any assumptions and simplifications made. | Yes. The list and definition are on Appendix 2. |
| Critical appraisal of individual sources of evidence§ | 12 | If done, provide a rationale for conducting a critical appraisal of included sources of evidence; describe the methods used and how this information was used in any data synthesis (if appropriate). | Critical appraisal of individual sources of evidence will not be done. |
| Synthesis of results | 13 | Describe the methods of handling and summarizing the data that were charted. | Yes. The methods of handling and summarizing the data that were charted are on page 8. |
| **RESULTS** | | | |
| Selection of sources of evidence | 14 | Give numbers of sources of evidence screened, assessed for eligibility, and included in the review, with reasons for exclusions at each stage, ideally using a flow diagram. | It's just the protocol, the study is not complete. |
| Characteristics of sources of evidence | 15 | For each source of evidence, present characteristics for which data were charted and provide the citations. | It's just the protocol, the study is not complete. |
| Critical appraisal within sources of evidence | 16 | If done, present data on critical appraisal of included sources of evidence (see item 12). | It's just the protocol, the study is not complete. |
| Results of individual sources of evidence | 17 | For each included source of evidence, present the relevant data that were charted that relate to the review questions and objectives. | It's just the protocol, the study is not complete. |
| Synthesis of results | 18 | Summarize and/or present the charting results as they relate to the review questions and objectives. | It's just the protocol, the study is not complete. |
| **DISCUSSION** | | | |
| Summary of evidence | 19 | Summarize the main results (including an overview of concepts, themes, and types of evidence available), link to the review questions and objectives, and consider the relevance to key groups. | It's just the protocol, the study is not complete. |
| Limitations | 20 | Discuss the limitations of the scoping review process. | It's just the protocol, the study is not complete. |
| Conclusions | 21 | Provide a general interpretation of the results with respect to the review questions and objectives, as well as potential implications and/or next steps. | It's just the protocol, the study is not complete. |
| **FUNDING** | | | |
| Funding | 22 | Describe sources of funding for the included sources of evidence, as well as sources of funding for the scoping review. Describe the role of the funders of the scoping review. | The financial disclosure is "JON received funds from the Graduate Support Program (PROAP/CAPES – Portaria  156 de 28 de novembro de 2014), through the for Edital DPG/UnB N 002/2021, for  english language editing of the manuscript. The funders had and will not have a role in  study design, data collection and analysis, decision to publish, or preparation of the  manuscript." |

JBI = Joanna Briggs Institute; PRISMA-ScR = Preferred Reporting Items for Systematic reviews and Meta-Analyses extension for Scoping Reviews.

* Where *sources of evidence* (see second footnote) are compiled from, such as bibliographic databases, social media platforms, and Web sites.

† A more inclusive/heterogeneous term used to account for the different types of evidence or data sources (e.g., quantitative and/or qualitative research, expert opinion, and policy documents) that may be eligible in a scoping review as opposed to only studies. This is not to be confused with *information sources* (see first footnote).

‡ The frameworks by Arksey and O’Malley (6) and Levac and colleagues (7) and the JBI guidance (4, 5) refer to the process of data extraction in a scoping review as data charting*.*

§ The process of systematically examining research evidence to assess its validity, results, and relevance before using it to inform a decision. This term is used for items 12 and 19 instead of "risk of bias" (which is more applicable to systematic reviews of interventions) to include and acknowledge the various sources of evidence that may be used in a scoping review (e.g., quantitative and/or qualitative research, expert opinion, and policy document).

*From:* Tricco AC, Lillie E, Zarin W, O'Brien KK, Colquhoun H, Levac D, et al. PRISMA Extension for Scoping Reviews (PRISMAScR): Checklist and Explanation. Ann Intern Med. 2018;169:467–473. [doi: 10.7326/M18-0850](http://annals.org/aim/fullarticle/2700389/prisma-extension-scoping-reviews-prisma-scr-checklist-explanation).
